# Snakebite prevalence and risk factors in a nomadic population in Samburu County, Kenya: A community-based survey

**DOI:** 10.1371/journal.pntd.0011678

**Published:** 2024-01-02

**Authors:** Frank-Leonel Tianyi, George O. Oluoch, Denis Otundo, Robert Ofwete, Cecilia Ngari, Anna Trelfa, Sayem Ahmed, Duolao Wang, Monica Smith, Viviane Meta, Nicholas R. Casewell, David G. Lalloo, Robert A. Harrison, Ymkje Stienstra

**Affiliations:** 1 Centre for Snakebite Research and Interventions, Liverpool School of Tropical Medicine, Pembroke Place, Liverpool, United Kingdom; 2 Department of Tropical Disease Biology, Liverpool School of Tropical Medicine, Pembroke Place, Liverpool, United Kingdom; 3 Kenya Snakebite Research and Intervention Centre, Kenya Institute of Primate Research, Ministry of Health, Karen, Nairobi, Kenya; 4 Health Economics and Health Technology Assessment, School of Health and Wellbeing, University of Glasgow, Glasgow, United Kingdom; 5 Department of Clinical Sciences, Liverpool School of Tropical Medicine, Pembroke Place, Liverpool, United Kingdom; 6 LocateIT Limited, Galana Road, Nairobi, Kenya; 7 University of Groningen, Department of Internal Medicine/Infectious Diseases, University Medical Centre Groningen, Groningen, The Netherlands; Fundação de Medicina Tropical Doutor Heitor Vieira Dourado, BRAZIL

## Abstract

**Introduction:**

Snakebite is an important public health concern, especially in tropical areas, but the true burden remains unclear due to sub-optimal reporting and over-reliance on health facility-based data.

**Methods:**

A community-based cross-sectional survey was conducted in Samburu County, Kenya from December 2019 to March 2020. Geospatial techniques were used to create a sampling frame of all households in Samburu County and a multistage cluster sampling strategy to select households and recruit study participants. Five year prevalence and mortality rates were estimated, the characteristics and circumstances of snakebite were described, and multilevel logistic regression models were built to identify independent risk factors for snakebite.

**Results:**

We recruited 3,610 individuals living in 875 households from 30 clusters. The 5-year prevalence of snakebite was 2.2% (95% CI 1.4%–3.4%), and the 5-year mortality rate was 138 (95% CI 44–322) deaths per 100,000 inhabitants, resulting in an estimated 1,406 snakebites and 88 deaths from snakebites per year in Samburu County. Snakebite incidents often occurred at night between 9pm and 6 am (44%, n = 36), and the participants were mostly walking/playing outdoors (51%, n = 41) or sleeping (32%, n = 27) when they were bitten. Lower household socioeconomic status and smaller numbers of people per house were significant independent risk factors.

**Conclusion:**

Samburu County has a high snakebite burden and the most victims are bitten while sleeping or walking outdoors at night. Snakebite prevention and health promotion programs in Samburu County, and other endemic regions, need to be contextualised and consider the geographic, seasonal, and temporal specificities found in our study. Our findings also have implications for health care delivery, especially identification of the need for night-time staffing with expertise in snakebite management and antivenom availability to better manage patients and thereby improve outcomes.

## Author summary

Contemporary reliable estimates of the number of cases and risk factors for snakebite are needed to inform interventions to reduce snakebite incidence, especially in countries with a high burden. We conducted a community-based study in Samburu County Kenya, using methods enabling the inclusion of mobile pastoralist communities that are hard-to-reach. Of the 3610 participants recruited into our study, 2.2% (95% CI 1.4%–3.4%) had experienced a snakebite in the past five years, and there were 138 (95% CI 44–322) deaths per 100,000 inhabitants. Snakebites were most common during the dry season and most victims were bitten when they were sleeping or walking/playing outdoors, usually at night. Living in a wealthier household or in a household with more inhabitants lowered the risk of a snakebite. Snakebite prevention interventions in Samburu County should consider the higher number of bites during the dry season and at night. Health facilities may need to ensure 24-hour availability of both antivenom treatment and of expertise in the management of snakebite.

## Introduction

Snakebite is an important public health concern, especially in tropical areas where a combination of environmental, geographical and demographic factors increases the chances of human-snake interactions [1]. Despite recent renewed global interest in snakebite, the true burden remains unclear. Sub-optimal reporting and data collection, over-reliance on health facility-based data and the production of modelled estimates from regional figures may have delayed the prioritization of snakebite as a major cause of death, and disability [1,2].

The estimated incidence of snakebites in the published literature varies widely in Africa (0.6–903 bites per 100,000 inhabitants), making it hard to guide national public health action against snakebite and to prioritize resources for prevention and treatment [3,4]. A facility-based study by Coombs et al. found an annual incidence of snakebite ranging from 1.9–67.9 per 100,000 inhabitants across counties in Kenya, with Samburu county being among the highest (66 per 100,000 inhabitants) [5]. Another community-based survey by Snow et al. showed a much higher annual incidence of snakebite in Kilifi County compared to the facility-based study by Coombs et al. (150 vs 44 per 100,000 inhabitants) [5,6]. The estimates of mortality were just as heterogenous, with a recurring theme of higher mortality estimates from the community-based survey, compared to the facility-based study (6.7 vs 0.45 snakebite-related deaths per 100,000 inhabitants) [5,6]. Both studies were conducted in the 90s and, given the structural and demographic changes that have occurred since then, their relevance to current health decision making is uncertain. Furthermore, the Northern-rift regions of Kenya are home to mobile pastoralist communities who may require different epidemiological approaches to those used in settled populations with fixed residences.

A retrospective, literature-based study based on data from 16 West African countries showed the burden of snakebite, expressed in disability adjusted life years (DALYs), was higher than that posed by other neglected tropical diseases in the region [7]. There is an urgent need for well-designed community-based studies to provide data on the true burden of snakebite in African regions and to identify factors which could predict the risk of snakebite, thereby informing preventive interventions and policy formulation.

To address these knowledge gaps, the objective of this study was to describe the health burden of snakebite by estimating the 5-year prevalence and mortality-rate from snakebite and identifying factors that may predict snakebite in Samburu County, Kenya. The study was undertaken as part of the multidisciplinary research objectives of the UK National Institutes of Health Research (NIHR) funded African Snakebite Research Group [8,9].

## Methods

### Ethics statement

Ethical approval for this survey was obtained from the Kenyatta National Hospital—University of Nairobi Ethics Research Council, Nairobi, Kenya (P195/04/2017) and the Liverpool School of Tropical Medicine Research Ethics Committee (Research Protocol 18–058). Written informed consent was obtained from household heads prior to starting the interview, and the household heads acted as proxy respondents for all household members. A witnessed verbal consent was obtained for household heads who could not read or write. Pseudonymised data was used for the analysis to ensure patient anonymity throughout the data analysis.

### Study setting

Samburu County is located in the Northern part of Kenya, about 300 km from the capital Nairobi, and lies in the northern part of the Great Rift Valley with altitudes ranging between 350 and 2,750 metres. Rainfall, vegetation, temperature and humidity indices show equally large variations. The county totalled 310,327 inhabitants in 2019, with a low population density (14.73 persons per km^2^), varying at the sub-county level from 8 to 29 persons per km^2^ in Samburu East and Samburu Central respectively [10]. Several pastoralist tribes reside in Samburu, and pastoralism drives the economy with 80% of the households keeping livestock. Pastoralist communities engage in transhumance activities where they frequently move their herds over large distances to find grazing land for their livestock. Samburu county had three hospitals in 2018: one county referral hospital and two sub-district level hospitals, 15 health centres, 59 dispensaries and 14 private clinics. Access to health facilities, especially hospitals, is limited by poor seasonal road networks, poverty, and regional insecurity resulting from inter-community livestock rustling [11].

### Sample size

This survey was designed to sample approximately 2% of the households in Samburu County. Using the number of households from the 2009 Kenyan Population and Housing Census, we calculated a sample size of 900 households. We adopted the definition of a household from the Kenyan Bureau of National Statistics which considers everyone who spent the previous night in the household as a member of the household. [12]. To minimise the effects of cluster bias in our sampling design and to accommodate funding and logistical constraints, we adopted a 36 x 25 design in which we selected 36 clusters in our first sampling stage, and 25 households from each cluster in our second sampling stage.

### Sampling strategy

A multistage cluster sampling strategy was employed, with a combination of random, geospatial and convenience methods. Geospatial techniques were used to digitize all homesteads (physical structures in which people live) in Samburu County from mid-August 2019 to mid-September 2019. The sampling frame consisted of homesteads identified by downloading, processing, and digitising high resolution satellite imagery from the Copernicus Sentinel-2 Earth observation mission of the European Space Agency that provides open-source high resolution optical imagery (10m spatial resolution) with 5-to-10-day revisits of the same location. The primary sampling units (PSU) were spatially generated 5-by-5-kilometre grids (clusters) covering the entire Samburu County. The cluster grids (956 grids) and the mapped households (50,707 homesteads) were merged to create a gridded household dataset containing the global positioning system (GPS) coordinates of each homestead. In this study, homesteads were physical structures that could be visualised spatially and geo-tagged, while households were social organisations with a single household head. We used homesteads as a proxy for households, and for the rest of the paper, we will use the word household to refer to both the physical structures and the social organisation. A probability proportional to size approach was used to select 36 grids with the sampling unit being a cluster-grid.

In the second sampling stage, a random number generator was used to select 25 households from each cluster, and the GPS coordinates were extracted and uploaded into navigator apps for the data collectors. The sampling and geo-tagging of selected clusters and households was completed in September 2019. A detailed description of the methods can be found in a separate publication [13].

### Data collection

Data collection took place between December 2019 and March 2020, with door-to-door visits by mobile teams made up of a trained data collector, a coordinator, and a local guide recruited from the cluster where the data was being collected. Interviewer administered questionnaires were used to collect sociodemographic household data, individual data on all household members, and details on snakebite incidents or deaths. Household heads, defined using standard Demographic Health Survey (DHS) criteria, acted as proxy respondents for all household members. Data was entered into GPS-enabled electronic tablets using the freely available KoboCollect app which securely encodes collected data and stores them locally and remotely with varying levels of restricted access. Data collection and analyses were delayed by COVID-19 travel and work restrictions.

### Data analysis

#### Important variables

Our main outcomes variables were the prevalence and mortality-rate of snakebite. We had initially intended to estimate the annual incidence and 5-year mortality of snakebite. However, during the study, we observed a discordance between the bites reported to have occurred in the past year, and the calculated difference in reported age at the time of the bite and the interview date in our data. Snakebite is a traumatic event that is susceptible to recall bias [14] and we found that whilst participants always recalled having a snakebite, the exact date or period when it happened was more difficult to ascertain. We thus adopted a 5-year prevalence approach instead of a 1-year prevalence which fixed the discordance between the reported age at the time of the bite, and the calculated age based on the interview date. We defined a snakebite case as a reported snakebite incident for which the participant provided answers to at least 90% of the questions on the circumstances and characteristics of the bite, health seeking behaviour, treatment received, and long-term complications after the bite, and for which the age difference on the day of the interview and at the time of the incident was not more than 5 years. We defined a snakebite death as a reported snakebite death for which answers were provided to at least 90% of the questions on the circumstances and characteristics of the bite, health seeking behaviour, treatment received after the bite and circumstances of the death. A list of the questions can be found in S1 Table.

The prevalence and mortality-rate of snakebite were estimated using the total number of snakebite cases and deaths respectively as numerators, and the total number of respondents in the study as the denominator. Sampling weights were applied, and 95% confidence intervals were estimated using DHS methodology [15].

Wealth index quintiles were used as a proxy for the socioeconomic status of households [16]. A principal component analysis was carried out using data on household assets and socioeconomic characteristics, and a wealth index score was calculated for each household. Households were categorised into quintiles with the lowest quintile (quintile 1) representing the poorest households, and the highest quintile (quintile 5) representing the richest households [17].

Descriptive analysis of cluster level, household level and participant-level data were carried out using proportions, means, and standard errors (SEs) for normally distributed variables and medians and interquartile ranges (IQRs) for nonparametric variables. Comparisons by presence or absence of a snakebite were made using chi-squared, Fisher’s exact, and Wilcoxon rank sum test as appropriate.

#### Multilevel regression analyses

Participants in this study were nested within households, which were in turn nested within clusters. This required us to use multilevel regression models to explore factors associated with snakebite in Samburu County. The analysis was adjusted for the clustered design (using the Stata *svyset* command) [18], and multilevel logistic regression models were fitted (using the Stata *melogit* command) [19] with occurrence of a snakebite as our outcome variable; wealth index, and number of household members as household-level explanatory variables; age and gender as individual-level explanatory variables; urban/rural location and sub-county as cluster-level explanatory variables; and random intercepts by cluster ID and household ID. Multivariable multilevel logistic regression models were built in three steps as outlined in Table 1 below [20]. Firstly, three empty models including the outcome variable and different combinations of the random intercepts (ClusterID alone, HouseholdID alone, both ClusterID and HouseholdID) were compared between each other and to a simple logistic regression model to justify the use of multilevel models in our analyses. We compared the estimates of the variance of the distribution of random effects, and the intracluster correlation coefficients (ICC) of each model. The ICC represents the proportion of the total observed individual variation in the outcome that is attributable to between-cluster variation, and it captures the within-cluster component of the individual variance and the between-cluster variation in each model. The second model involving only the household-level random intercept failed to converge (only two (0.2%) households had more than one snakebite victim). For the rest of our analysis, we assumed that household-level and individual-level variables carried the same risk of snakebite (level 1), and that they were nested within clusters (level 2). We retained model 1 which included the random effects at cluster level, and this was used as our base model for the rest of the analysis. In the second step, two models were built by combining individual-level and household-level variables to assess individual-level effects, and by using only cluster-level explanatory variables in the second model to assess area-level effects. In the final step, all three groups of the explanatory variables were added to the model.

**Table 1:** Contents and description of multilevel models used in our analyses.

Models	Level	Fixed effects variables	Random effects variables
Model 1	-	-	ClusterID
Model 2	Individual-level and household-level	wealth index, number of household members; age and gender	ClusterID
Model 3	Individual-level, household-level and cluster-level	wealth index, number of household members, age, gender urban/rural location and sub-county	ClusterID

Adjusted odds ratios (aORs) were estimated to assess the relationship between the explanatory and the outcome variables, accounting for random effects at the cluster level and for clustering. Details of the model specification can be found in S2 Table.

The models, which included cluster-level explanatory variables, had an increased risk of potential overlapping effects due to potential interactions between the area level variance and the residual effects of the random intercepts. In addition to using the ICC to quantify the variance of random effects, we also estimated the median odds ratio (MOR) to quantify and compare cluster-level effects, the 80% interval odds ratio (IOR) to quantify and compare the combined effects of cluster-level variance and residual variability across clusters, and the proportion of opposed odds ratios (POOR) which compares the proportion of individual pair-wise odds ratios with an effect in the opposite direction to the overall cluster-level odds ratio [20]. An IOR which contains the null value (1) suggest a large residual cluster-variance compared to the variability of the cluster-level variable. A small POOR (0–50%) suggests homogeneity, meanwhile a larger POOR (50–100%) suggests heterogeneity between the cluster-level and residual effects [20]. Finally, we calculated the proportional change of variance for each model, using the previous nested model as a reference.

For models which contained individual-level risk factors, we also estimated the specific IOR to compare the residual cluster variance to the variability of the individual-level variables. Model fitness was assessed by comparing the predicted and observed values using the *predict* postestimation command. We also calculated the area under the curve (AUC) to measure and compare the ability of our models to correctly classify individuals with or without a snakebite by plotting the true positive fraction against the false negative fraction for different binary classification thresholds of the predicted probabilities. The AUC ranged from 0.5 to 1, with an AUC of 1 indicating a perfect discrimination, while an AUC of 0.5 suggested no predictive power. Two-sided p-values < 0.05 were taken to indicate statistical significance. Analysis was conducted using Stata 16.1 (StataCorp LP, College Station, TX, USA).

## Results

### Individual and household sociodemographic data

We collected data from 3,610 individuals living in 875 households from 30 clusters, with a response rate of 98.4%. Because our survey included nomadic pastoralist communities, only 409 (46.7%) households were in the same geo-location as the initial geospatial mapping exercise (conducted three months prior to the survey). In six clusters, all the inhabitants had abandoned their homesteads and migrated to a different location, some as part of their usual transhumance movements, and others due to inter-tribal conflicts and insecurity.

There was a slightly higher number of female inhabitants (51.1%), and the 0–10 years age category comprised the highest proportion (34.6%) of participants. Most households were in rural areas (88.8%) and one in four household heads had no formal education. The main individual and household sociodemographic characteristics of the participants are presented in Table 2.

**Table 2:** Household level and participant-level socioeconomic data stratified by households that did or did not report a snakebite.

Socioeconomic variables	No snakebite (%)	Snakebite (%)	Total (%)	p-value^
**Cluster level variables**				
Sub-county				0.010
Samburu east	2 (28.6%)	9 (39.1%)	11 (36.7%)	
Samburu north	2 (28.6%)	13 (56.5%)	15 (50.0%)	
Samburu central	3 (42.8%)	1 (4.4%)	4 (13.3%)	
Urban/rural location				0.003
Urban	3 (42.8%)	1 (4.4%)	4 (13.3%)	
Rural	4 (57.2%)	22 (95.6%)	26 (86.7%)	
Cluster Total	7	23	30	
**Household level variables**				
Marital status of household head				0.86
Single	389 (48.9%)	41 (51.9%)	430 (49.1%)	
Married	298 (37.4%)	28 (35.4%)	326 (37.3%)	
Widowed/Divorced/Separated/Others	109 (13.7%)	10 (12.7%)	119 (13.6%)	
Educational status of household head				<0.001
No formal education	636 (79.9%)	56 (70.9%)	692 (79.0%)	
Primary School	14 (1.8%)	4 (5.1%)	18 (2.0%)	
Secondary School	90 (11.3%)	16 (20.2%)	106 (12.5%)	
Tertiary/post-secondary education	56 (7.0%)	3 (3.8%)	59 (6.5%)	
Household health insurance				0.87
No known health insurance	743 (93.3%)	74 (93.7%)	817 (93.4%)	
Health insurance	53 (6.7%)	5 (6.3%)	58 (6.6%)	
Quintiles of wealth index				0.20
Poorest	156 (19.5%)	21 (26.6%)	177 (20.2%)	
Second	163 (20.5%)	17 (21.5%)	180 (20.5%)	
Third	162 (20.4%)	11 (13.9%)	173 (19.8%)	
Fourth	155 (19.5%)	21 (26.6%)	176 (20.1%)	
Richest	160 (20.1%)	9 (11.4%)	169 (19.4%)	
Household Total	796	79	875	
**Participant level variables**				
Age, median (IQR)	17.0 (7.0–32.0)	27.0 (16.0–46.0)	17.0 (7.0–32.0)	<0.001*
Gender				0.97
Female	1,802 (51.1%)	41 (50.6%)	1,843 (51.1%)	
Male	1,727 (48.9%)	40 (49.3%)	1,767 (48.9%)	
People per household, median (IQR)	5.0 (4.0–6.0)	4.0 (2.0–5.0)	5.0 (4.0–6.0)	<0.001*
Total	3,529	81	3,610	

^Chi-squared p-values comparing binary/categorical cluster-level, household level and individual-level socioeconomic factors by presence or absence of a snakebite at the respective level;

*Wilcoxon rank sum test comparing continuous participant level socioeconomic variables by presence or absence of a snakebite.

### Prevalence and mortality-rate of snakebite

**Fig 1:**
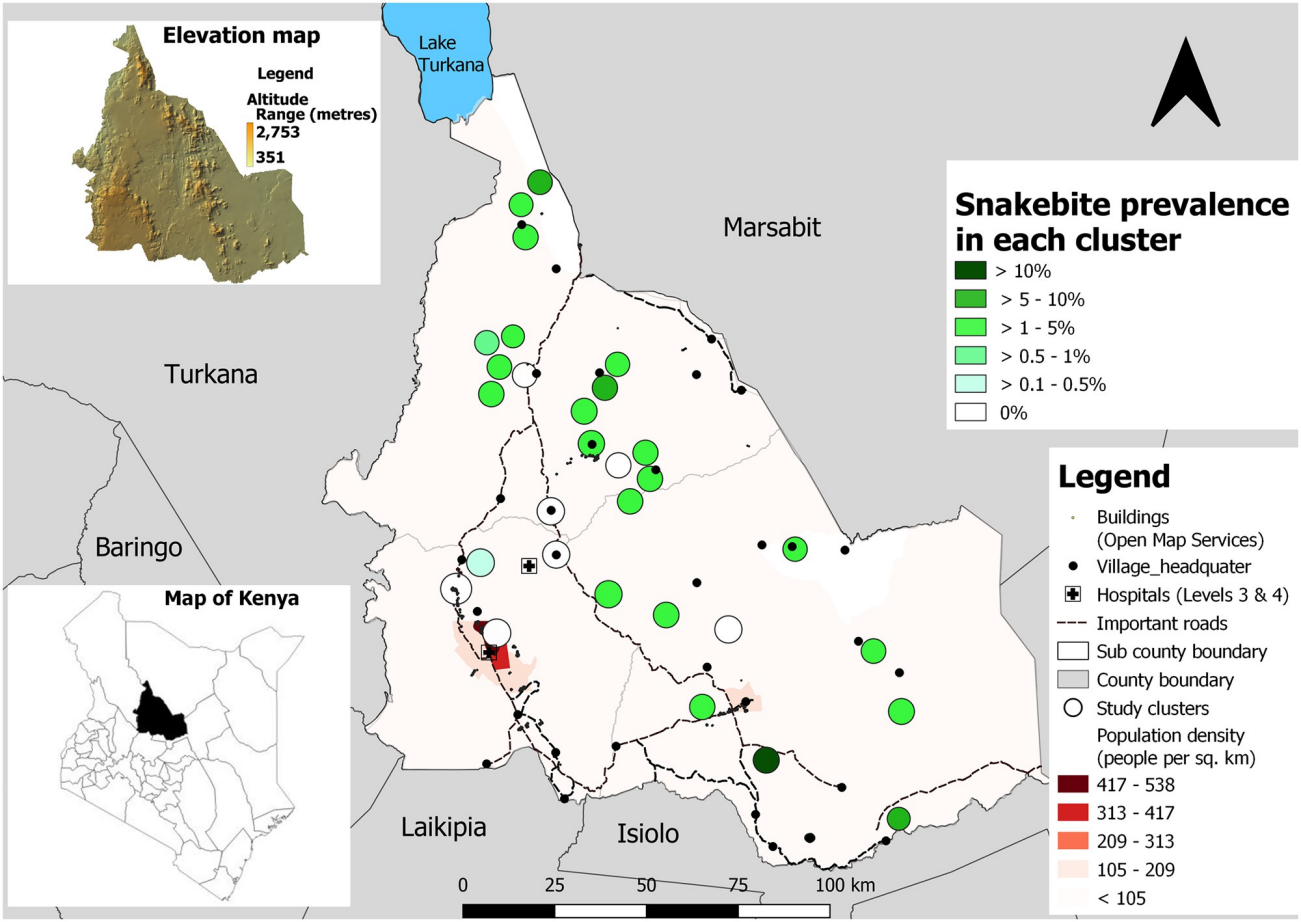
Prevalence of snakebite in study clusters with population density and elevation in Samburu County, Kenya *(Created using the Free and Open Source QGIS v3*.*22*.*3*, *base map obtained from* https://data.humdata.org/).

In our study population, we identified 81 snakebite victims and five snakebite-related deaths, in geographically distinct locations, that occurred within the past 5 years. The 5-year prevalence of snakebite was 2.2% (95% CI 1.4%–3.4%), and the 5-year mortality rate was 138 (95% CI 44–322) deaths per 100,000 inhabitants. There was a considerable variation in snakebite prevalence with seven (23%) clusters reporting no snakebites, and the 5-year prevalence of snakebite varying between 0.4% and 11.2%. All clusters in densely populated areas or along major roads reported no snakebites (see Fig 1). There was no significant difference in gender-specific prevalence in our study population.

### Circumstances and characteristics of snakebite

The median age when snakebites occurred was 27.0 (IQR; 16–45.0) and there was no significant difference in prevalence between male (49.3%, n = 40) and female (50.6%, n = 41) victims. Snakebites often occurred at night between 9pm and 6 am (44%, n = 36), and the participants were mostly walking/playing outdoors (51%, n = 41) or sleeping (32%, n = 27) when they were bitten. Snakebites were most common during the dry season (58%, n = 47), with a peak in snakebites cases between the months of December and January (S1 Fig).

### Health seeking behaviours, treatment received and complications

Treatment was most frequently first sought at a dispensary (34.6%, n = 28) following a snakebite, and only a small proportion of participants went to a hospital (11.1%, n = 9), with some not seeking any treatment at all (12.3%, n = 10) while others sought treatment from a traditional healer (22.2%, n = 18).

For participants who sought treatment (initial and subsequent treatment locations) at a health facility (n = 56), most arrived on the same day the bite occurred (78.6%, n = 44), and motorbikes were the most common form of transportation (50%, n = 28). Details of the snakebite incident and treatment received as reported by our study participants can be found in S3 and S4 Tables.

All participants, irrespective of their health seeking behaviour, were, according to the household head, still affected after the snakebite incident, with long-term pain (70.4%, n = 57) and low mood and/or anxiety (19.8%, n = 16) being the most commonly mentioned sequelae. Ten (17.9%) patients reported they required surgery and one (1.2%) required an amputation.

### Factors associated with snakebite

The multilevel logistic regression models enabled a separation of the effects associated with snakebite into (i) general and (ii) specific cluster-level effects, and then into (iii) general and (iv) specific individual-level effects and Table 3 presents a summary of these effects. We selected household wealth index, number of household members, participant age and gender, urban/rural location, and sub-county as *a priori* explanatory variables for our multilevel logistic regression analysis. There was a high degree of multicollinearity between urban/rural location and sub-county (regression coefficient r = 0.7), hence only sub-county was retained for multivariable analysis.

**Table 3:** Factors associated with a snakebite from multivariable analysis: Estimated Odds Ratios from multilevel models.

Variables	Step 1	Step 2		Step 3
	Empty model	Model 1 Individual-level variables OR (95% CI)	Model 2 Cluster-level variable OR (95% CI)	Model 3 Combined variables OR (95% CI)
**General individual-level effects**				
Age category				
0–10		ref		ref
11–20		2.30 (0.97–5.45)		2.30 (0.97–5.45)
21–30		2.35 (0.92–5.97)		2.35 (0.92–5.97)
31–40		2.17 (0.87–5.42)		2.17 (0.87–5.41)
41–50		3.15 (0.89–11.07)		3.15 (0.89–11.07)
>51		2.82 (0.79–10.04)		2.82 (0.79–10.04)
Gender				
Female		ref		ref
Male		1.22 (0.78–1.90)		1.22 (0.78–1.90)
People per household		0.69 (0.53–0.90)		0.69 (0.53–0.90) **
Wealth index				
First quintile (poorest)		1.2 (0.52–2.72)		1.18 (0.52–2.70)
Second quintile		4.29 (1.81–10.17)		4.26 (1.80–10.10) **
Third quintile		3.50 (1.63–7.51)		3.48 (1.63–7.42) **
Fourth quintile		2.11 (0.75–5.91)		2.11 (0.75–5.90)
Fifth quintile (richest)		ref		ref
Sub-County				
Samburu east/central			ref	ref
Samburu north			1.83 (0.38–8.71)	1.51 (0.34–6.69)
**Specific Individual-level estimated effects 80% IOR (Lower IOR—Upper IOR)**				
Oldest vs youngest age-groups		0.33–24.18		0.33–24.28
Male vs female		0.14–10.43		0.14–10.47
Poorest vs richest wealth quintiles		0.12–8.57		0.12–6.61
People per household		0.08–5.90		0.08–5.92
**Specific cluster-level estimated effects**				
Samburu North vs East/Central [OR (95%CI)]			1.83 (-0.91–4.56)	1.51 (-0.64–3.66)
80% IOR (lower IOR—upper IOR)			0.18–18.43	0.18–13.00
POOR (%)			0.37	0.40
**General cluster-level effects**				
Cluster variance	1.66	1.40	1.62	1.41
PCV (%)	-	-16%	-2%	13%
ICC (%)	0.34	0.29	0.33	0.30
MOR	3.42	3.10	3.38	3.10
AUC (95%CI)	0.74 (0.69–0.79)	0.82 (0.77–0.86)	0.74 (0.69–0.79)	0.82(0.77–0.86)
AUC change		0.07	0.00	0.07

ICC, intraclass correlation coefficient; IOR, interval odds ratio; MOR, median OR; PCV, proportional change of the variance; POOR, the proportion of OR in the opposite direction; AUC, area under the receiver operating characteristic curve; ref, reference category.

Individual-level variables: wealth index, number of household members, age categories, gender Cluster-level variable: sub-county

Combined variables: wealth index, number of household members, age categories, gender, and sub-county

*p-value < 0.05;

**p-value < 0.01;

***p-value < 0.001

### General cluster-level effects

The general cluster-level effects were estimated using a 3-step process to extract and compare variance components. In step 1 (empty model), 34% of the total variance in the odds of snakebite was attributed to between-cluster variation. The between-cluster variance declined progressively in the next two steps, with an ICC of 33% in model 1 (individual-level variables) of step 2 and 30% in step 3 (combined individual and cluster-level variables). The residual cluster variance in the empty model was 166% and adding both individual-level and cluster-level variables resulted in a residual cluster-variance of 141%, suggesting that 15% of the predicted outcome of snakebite could be explained by the reported individual-level and cluster-level variables. The median increased odds of having a snakebite for an individual moving from a low-risk to a high-risk cluster was 3.40 in the empty model and 3.10 in the combined model. Measuring the AUC using the empty model was 0.74 [95% Confidence Interval (95% CI): 0.69–0.79] and adding the individual-level and cluster-level variables in step 3 increased the AUC by 0.07 units.

### General individual-level effects

The estimated individual-level effects in our final model all included the null value, indicating a large difference between the residual cluster variance and the individual-level variance and suggesting that the unmeasured risk factors of snakebite at the cluster level may contribute significantly to the risk of snakebite (see Table 3).

### Specific cluster-level effects

After adjusting for individual-level and cluster-level variables, and for the cluster-random effects, there was no significant association between subcounty location and snakebite risk. Comparing the contributions of the estimated variance of the sub-county location and the residual cluster-level variance showed a large difference between the two (IOR: 0.18–13.0), and in 40% of pairwise comparisons, the effect was in opposite directions (see Table 3).

### Specific individual-level effects

After adjusting for individual-level and cluster-level variables, and for the cluster-random effects, there was no significant association between age and gender, and snakebite risk. A unit increase in the number of people per household were associated with a 31% decrease in the odds of snakebite (OR: 0.69, 95% CI: 0.53–0.90). Compared to participants in the richest (fifth) wealth quintile, individuals in the second and third quintiles had 4.26 (95% CI: 1.80–10.10) and 3.48 (95% CI: 1.63–7.42) higher odds of snakebite.

## Discussion

### Prevalence and mortality from snakebite

This study reported a 5-year prevalence of snakebite of 2.2% (~ 440 per 100,000 inhabitants per year), and a 5-year mortality rate of 138 deaths per 100,000 inhabitants (~ 27.6 per year) respectively. There was no significant difference by gender, and the prevalence of snakebite increased by age categories, but with wide confidence intervals. Applying these rates to the whole population of Samburu results in an estimated 1,406 snakebites and 88 deaths from snakebites per year.

These figures are higher than previous studies in Kenya (440 vs 150 snakebites per 100,000) [5,6] but are consistent with contemporary evidence of a much higher incidence from community-based surveys, compared to hospital-based studies [1,21–23]. Although a difference of up to 60% between incidence values from hospital-based and community-based records has been reported, the authors of the hospital-based study acknowledged that their approach may have underestimated the true incidence in communities of Samburu [5,22]. Our estimates are lower than those in Akonolinga, Cameroon (600 per 100,000) [21]; Phin, Laos (1105 per 100,000) [24], but higher than those in Senegal (92 per 100,000) [25] and Nepal (251 per 100,000) [26]. Snakebite surveys are particularly susceptible to study site selection bias, especially when they are conducted at sub-national levels. Researchers often select study sites based on accessibility or prior knowledge of high snakebite incidence, and this could explain the heterogeneity in studies reporting snakebite prevalence or incidence [3]. The most robust estimates usually derive from well-designed nationwide surveys as reported in Nepal (251 per 100,000) [26] and Sri Lanka (391 per 100,000) [22], but these surveys require substantial funds and resources. Furthermore, our finding of a discrepancy between bites reported to have occurred in the past year, and the difference in age at the time of the bite and the interview date may also explain the heterogeneity in previous estimates and may be of interest to researchers conducting incidence studies. We recommend validating all reported snakebite cases to ensure an accurate numerator and more reliable estimates.

### Circumstances and characteristics of snakebite

We found that the 21–30-year age group had the highest proportion of bites, which is consistent with findings from prior studies in Kenya and other countries in sub-Saharan African (SSA) [6,27,28]. However, this association did not persist in our multilevel regression analysis. We did not find a gender difference in the risk of having a snakebite, contrary to findings in other SSA countries such as Nigeria and Cameroon where males were more likely to have a snakebite [21,29]. This may reflect cultural differences in gender-roles. A similar study in Bangladesh found no gender-difference in the risk of snakebites [30]. They suggested that the community-based study design eliminates gender-based differences in healthcare seeking behaviours which could explain the differences observed in bite incidence between males and females in other studies [21,28–30].

Snakebites in our study often occurred at night and the two most common activities that resulted in a bite were walking/playing outdoors and sleeping. Similar findings were found by previous studies in Kenya, and other countries in SSA and Asia [5,6,28,30]. At night, especially during very dry periods, snakes can venture into homes in search of prey or water. Some elapid species such as spitting cobras are most active at night and usually found close to homesteads in holes or bushes [28,31]. The way most houses in these rural communities are constructed makes it easy for snakes to get in at night and to bite victims in their sleep. This is typical of the snake species *Naja pallida*, commonly known as the ‘red spitting cobra’, which is commonly found in North Eastern Kenya [31,32]. Further, most of these houses have external toilets with poor lighting, making it easy to step on a snake while going to use the toilet at night. The low number of bites that occurred during farming and herding activities suggest that unlike in North Eastern Nigeria where snakebite is an occupational agricultural hazard [23,33], snakebites in Samburu county are less predictable and occurred outdoors, indoors and even while victims were asleep.

The seasonal variation in snakebite (S1 Fig) was consistent with a previous study in Kenya [5], with most bites occurring during the dry and hot months of June through to September, January and February. Samburu county is located in the arid and semi-arid lands (ASAL) of Kenya, with limited amounts of rainfall, which precludes a correlation between snakebite and rainfall as has been observed in previous studies [34,35].

### Health seeking behaviour, treatment and complications

Sixty-six percent (53/81) of victims first sought treatment at a health facility after a bite as reported by the household head. It is worth noting that 53% (28/53) of these victims went to a dispensary, which, because they are not staffed by doctors, nor equipped to treat snakebite victims, most frequently serve as the first point of referral in a snakebite victim’s complex pathway to treatment.

In our study a much smaller proportion of victims (22%) first sought treatment of a traditional healer compared to the 1994 study by Snow et al (59%) [6]. Also, most victims (77%) arrived at the health facility on the same day as the snakebite. Previous studies reported a preference of snakebite victims to consult traditional healers rather than hospital care and a prolonged delay in reaching health facilities [5,6]. Our findings may indicate changed health seeking behaviour in recent decades, similar to findings on decision making in health seeking from a qualitative study in Kenya [36], and from community-based surveys in Asia [37,38]. We also recognise the possible contribution of the numerous (i) community-engagement and (ii) social and news media events conducted by the Kenya-Snakebite Research & Intervention Centre team to raise awareness on the benefits of accessing hospital care following a snakebite [9].

Further research centred around decision making in health seeking behaviour after a snakebite is needed to confirm the data and to better understand barriers and facilitators in the decision making, especially given the differences in social organisation and livelihood of pastoralist communities compared to sedentary populations. Only one case of an amputation was reported. Long-term pain and low mood/anxiety were the most frequently reported long term complications of the bite. The frequently mentioned long-term pain by many of our study participants requires further analysis, including the specific type of pain reported, as long-term regional pain syndromes are rarely reported as a complication of snakebite envenoming [39,40]. The often-mentioned low mood and anxiety warrants a more detailed and validated assessment of mental health after snakebite envenoming.

To establish sustained improvement in the outcomes for tropical snakebite victims, there is an urgent need to better understand the long term physical, psychological and socioeconomic burdens of snakebite—ideally disaggregated by snake species, clinical syndrome, accessibility to health care and geography [23,41]. This improved understanding of snakebite burden will help guide the design of snakebite management policy and garner support from key national and international stakeholders.

### Factors associated with snakebite

After adjusting for recorded individual- and cluster-level variables, there was an association between the number of people per household and the household wealth index, with a 31% decrease in the odds of snakebite for each additional household member, and poorer households having higher odds of snakebite (up to four times higher) compared to the wealthiest households. This decrease in odds for each additional household member suggests a change in human-snake interaction based on household size eg due to differences in housing conditions or activities of household members.

The use of multilevel regression models enabled a partitioning of the contribution of recorded risk-factors to the risk of snakebite. Our analysis showed that a significant proportion of risk of snakebite was unaccounted for by our data. There was a strong geographic component to the risk of snakebite, with the effect of clustering dominating the effect of individual and cluster-level risk factors. These also suggest an environmental contribution to the risk of snakebite envenoming, which may not have been properly accounted for in our study. Our study used a geospatial sampling technique with a random selection of grids and households, hence we were unable to find specific geographic data on snake species and activity, and environmental data that corresponded to our selected clusters.

Nonetheless, our results support the hypothesis of a mechanistic model of snakebite, arising from the product of human and snake abundance, with combinations of human factors (social, economic and socioeconomic status), snake factors (activity, behaviour, human impact) and environmental factors (climate, landcover and topography) [42]. The link between geography and snakebite has been made by prior studies, with risk factors such as an abundance of natural habitats for snakes, low human population density in rural areas, occupational determinants, and high proportions of the population belonging to at-risk population groups for snakebites [1,4,5,23,28,43]. This further emphasises the complexity in predicting snakebites, and the need for cross-disciplinary collaborations [44] to increase chances of significantly reducing the burden of snakebite by 2030—a WHO priority [45].

### Study strengths and limitations

To our knowledge, this is the first comprehensive community-based survey of the burden of snakebite in Samburu County. The strengths of our study lie in the fact that we combined geospatial and random sampling techniques to create a sampling frame enabling participation from a mobile nomadic population. We also validated each snakebite case, relying not only on the response to our questionnaires, but ensuring consistency with participant age. Finally, we conducted a multilevel analysis to incorporate our complex sampling design and better understand the relationship between geography and snakebite. Nonetheless, our findings should be considered with the following limitations in mind. Our data depended on the recall of participants, with a potential of recall bias, especially regarding the details of snakebite incidents, and in 50% of cases, this information was not provided directly by the victim themselves, but by the household head. Our inability to collect data on snake-related and environmental factors may have led to residual confounding in our association analysis.

## Conclusion

Samburu County has a high snakebite burden with an estimated 1,406 snakebites and 88 deaths from snakebites per year. Most bites occur during the dry and hot months, with a peak in January, and bites were most frequent at night when victims were walking/playing outdoors or sleeping indoors.

In Samburu, more people per house and higher household wealth lowered the risk of snakebite, and snakebite risk was strongly linked to the geographic location of sampled clusters. Snakebite prevention and health promotion programs in Samburu County need to consider these geographic, seasonal, and temporal specificities. Our findings also have consequences for health care delivery, especially staffing and antivenom availability. Health facilities may need to ensure 24-hour availability of antivenoms and of clinical expertise in the management of snakebite, especially in endemic areas.

## Supporting information

S1 Table Questionnaire items on snakebite cases and deaths. (DOCX)

S2 Table Details of multilevel regression analyses. (DOCX)

S3 Table Details of the snakebite incident for participants who reported a snakebite. (DOCX)

S4 Table Treatment details reported by participants who sought treatment at a health facility after a snakebite. (DOCX)

S1 Fig Monthly variation in snakebite cases in Samburu County. (TIF)
