# Supplementary material for: Snakebite prevalence and risk factors in a nomadic population in Samburu County, Kenya: A community-based survey
Source: PLoS Negl Trop Dis. 2024 Jan 2;18(1):e0011678. doi: 10.1371/journal.pntd.0011678 (PMC10760648; doi:10.1371/journal.pntd.0011678)
Supplement: S1 Table — (DOCX) [file pntd.0011678.s001.docx]

**S1Table. Questionnaire items on snakebite cases and deaths**

| **Snakebite Cases – Questions**  How old was the individual when the snakebite occurred?  In what month did the snakebite occur?  What time of the day did the snakebite occur?  What were they doing at the time of the snakebite?  Where on their body was the snakebite?  Was the individual covered by any form of health insurance, at the time of the snakebite?  Where was the ﬁrst place of treatment, immediately after the snakebite?  Costs for ﬁrst place of treatment ?  Did they seek further help after the ﬁrst place of treatment?  If the individual attended hospital for their snakebite, what was the main mode of transport they used to get to hospital?  How long did it take them to arrive at the hospital after the snakebite?  How long did it take them to arrive at the hospital after the snakebite?  How many days did they stay in hospital?  Did they undergo surgery after the snakebite?  Did any of the following happen to them during their hospital stay?  After leaving the hospital, did they attend any other places to receive treatment in the ﬁrst 7 days?  Did they receive antivenom at their place of treatment?  How have they been aﬀected after the snakebite?  Did the eﬀects of the snakebite impact on their ability to perform their normal day-to-day activities?  If the snakebite did impact on their day-to-day activities, for how many weeks were they not able to perform their normal day-to-day activities (eg. work, home or school duties)?  Did the eﬀects of the snakebite impact on their social life?  If the snakebite did impact on their social life, for how many weeks were they unable to return to their normal social duties?  What is your (the patient) occupational status/ What is your (the patient) current stage of education?  How many days were you (the patient) unable to work/do housework/ How many days were you (the patient) absent from school?  What is your (the patient) daily wage? (KSH)  So, since returning home from any initial treatment, how many further visits have they made for treatment?  Please record the TOTAL AMOUNT spent on each of the following during all of their further visits for treatment (KSH): |
| --- |
| **Snakebite deaths - Questions**  How many deaths due to snakebite have occurred in this household in the last ﬁve years?  What was the relationship of the deceased individual to the respondent?  How old were they when they died?  What was the sex of the deceased?  Where was the ﬁrst place of treatment?  If the deceased individual attended hospital for their snakebite, what was the main mode of transport they used to get to hospital?  How long did it take them to travel to the hospital after the snakebite?  Did they receive antivenom after the snakebite?  How soon after the snakebite did they die?  Where did they die? |
