# Supplementary material for: Snakebite prevalence and risk factors in a nomadic population in Samburu County, Kenya: A community-based survey
Source: PLoS Negl Trop Dis. 2024 Jan 2;18(1):e0011678. doi: 10.1371/journal.pntd.0011678 (PMC10760648; doi:10.1371/journal.pntd.0011678)
Supplement: S2 Table — (DOCX) [file pntd.0011678.s002.docx]

**S2 Table.** *Details of multilevel regression analyses*

| The models were specified as:  = *β_0_ + β_1_*$x$*_1, i_ + β_2_*$x$*_2, i j_+ β_3_* $x$*_3, i j_ + ƅ_i_* ^(1)^ + Ɛ_i j_  log $\left( \begin{aligned} \frac{\text{ µ}\text{i}\text{ j }}{1- \text{µ}\text{i}\text{ j}} \\ \end{aligned} \right)$    (I)  Logit ($\text{Y}\text{i }\text{j }$= 1) =  (II)  *µ _i j_ = Pr* $\left( \text{Y}\text{i j }\text{ = 1}\text{ } \vert x\text{i j }\text{, }\text{ƅ}\text{i}\text{ }\text{(1)} \right)$ *ƅ_i_* ^(1)^ *~ Ɲ (0, δ^2^_1_)*  where $\text{Y}\text{i}\text{ j}\text{ }$ is the binary dependent variable with a value of 0 (the individual did not have a snakebite) or 1 (the individual had a snakebite) for the j^th^ individual within the i^th^ cluster; $x$*_i_* represents cluster-level explanatory variables; *µ _i j_* is the prevalence (risk) of a snakebite ( $\text{Y}\text{i j }$ = 1) given specific values of $x,$ *ƅ_i_ ^(1)^* ; $x$*_i j_* represents individual-level explanatory variables; *β* is the correlation coefficient (fixed effects) for each explanatory variable*;*  *ƅ_i_* ^(1)^ *~ Ɲ (0, δ^2^_1_)* is a random effect estimate for each cluster that accounts for cluster-specific variations in effect estimates; and Ɛ_i j_ is an error term. |
| --- |
