# Supplementary material for: Snakebite prevalence and risk factors in a nomadic population in Samburu County, Kenya: A community-based survey
Source: PLoS Negl Trop Dis. 2024 Jan 2;18(1):e0011678. doi: 10.1371/journal.pntd.0011678 (PMC10760648; doi:10.1371/journal.pntd.0011678)
Supplement: S3 Table — (DOCX) [file pntd.0011678.s003.docx]

S3 Table. Details of the snakebite incident for participants who reported a snakebite

| **Snakebite details** | **Frequency (n, %)** |
| --- | --- |
|  |  |
| Age at time of bite, median (IQR) | 27.0 (16.0-45.0) years |
| Age categories |  |
| 0-10 | 10 (12%) |
| 11-20 | 17 (21%) |
| 21-30 | 21 (26%) |
| 31-40 | 12 (15%) |
| 41-50 | 9 (11%) |
| >51 | 12 (15%) |
| Time of bite |  |
| Morning 6am - 12pm | 8 (10%) |
| Afternoon 12pm - 6pm | 16 (20%) |
| Evening 6pm - 9pm | 21 (26%) |
| Night 9pm - 6am | 36 (44%) |
| Season of bite |  |
| Dry season (Jan, Feb, Jun, July, Aug, Sept) | 47 (58%) |
| Long Rainy season (March, April, May) | 10 (12%) |
| Short rainy season (Oct, Nov, Dec) | 20 (25%) |
| Unable to recall | 4 (5%) |
| Activity at time of bite |  |
| Herding | 3 (4%) |
| Farming | 2 (2%) |
| Other indoor activity - cooking/playing | 8 (10%) |
| Other outdoor activity - walking/playing | 41 (51%) |
| Sleeping | 27 (33%) |
| Bite Site |  |
| Arms and shoulder | 4 (5%) |
| Hands and forearm | 9 (11%) |
| Ankle and foot | 34 (42%) |
| Head or neck | 6 (7%) |
| Leg above knee | 4 (5%) |
| Leg between ankle and knee | 14 (17%) |
| Spit in the eyes/body | 5 (6%) |
| Trunk | 3 (4%) |
| Cannot recall | 2 (2%) |
| Total | 81 |
