# Supplementary material for: Snakebite prevalence and risk factors in a nomadic population in Samburu County, Kenya: A community-based survey
Source: PLoS Negl Trop Dis. 2024 Jan 2;18(1):e0011678. doi: 10.1371/journal.pntd.0011678 (PMC10760648; doi:10.1371/journal.pntd.0011678)
Supplement: S4 Table — (DOCX) [file pntd.0011678.s004.docx]

S4 Table. Treatment details reported by participants who sought treatment at a health facility after a snakebite

| **Treatment details** | **Frequency (n, %)** |
| --- | --- |
|  |  |
| Transportation to health facility |  |
| By foot | 10 (17.9%) |
| Helicopter | 1 (1.8%) |
| Motorbike | 28 (50%) |
| Car/Taxi | 13 (23.2%) |
| Ambulance | 3 (5.4%) |
| Do not know | 1 (1.8%) |
| Delay in health facility presentation |  |
| Same day | 44 (78.6%) |
| Next day | 6 (10.7%) |
| >2 days | 5 (8.9%) |
| Do not recall | 1 (1.8%) |
| Duration of admission, median (IQR) | 1 (1 – 3); max 60 days |
| Had surgery |  |
| Yes | 10 (17.9%) |
| Complication during admission |  |
| Wound infection | 15 (26.8%) |
| Other infection | 3 (5.4%) |
| No complication | 38 (67.9%) |
| Reported antivenom use |  |
| Yes | 50 (89.3%) |
|  |  |
| Total | 56 |
