# Supplementary figures and images for: Snakebite prevalence and risk factors in a nomadic population in Samburu County, Kenya: A community-based survey
Source: PLoS Negl Trop Dis. 2024 Jan 2;18(1):e0011678. doi: 10.1371/journal.pntd.0011678 (PMC10760648; doi:10.1371/journal.pntd.0011678)

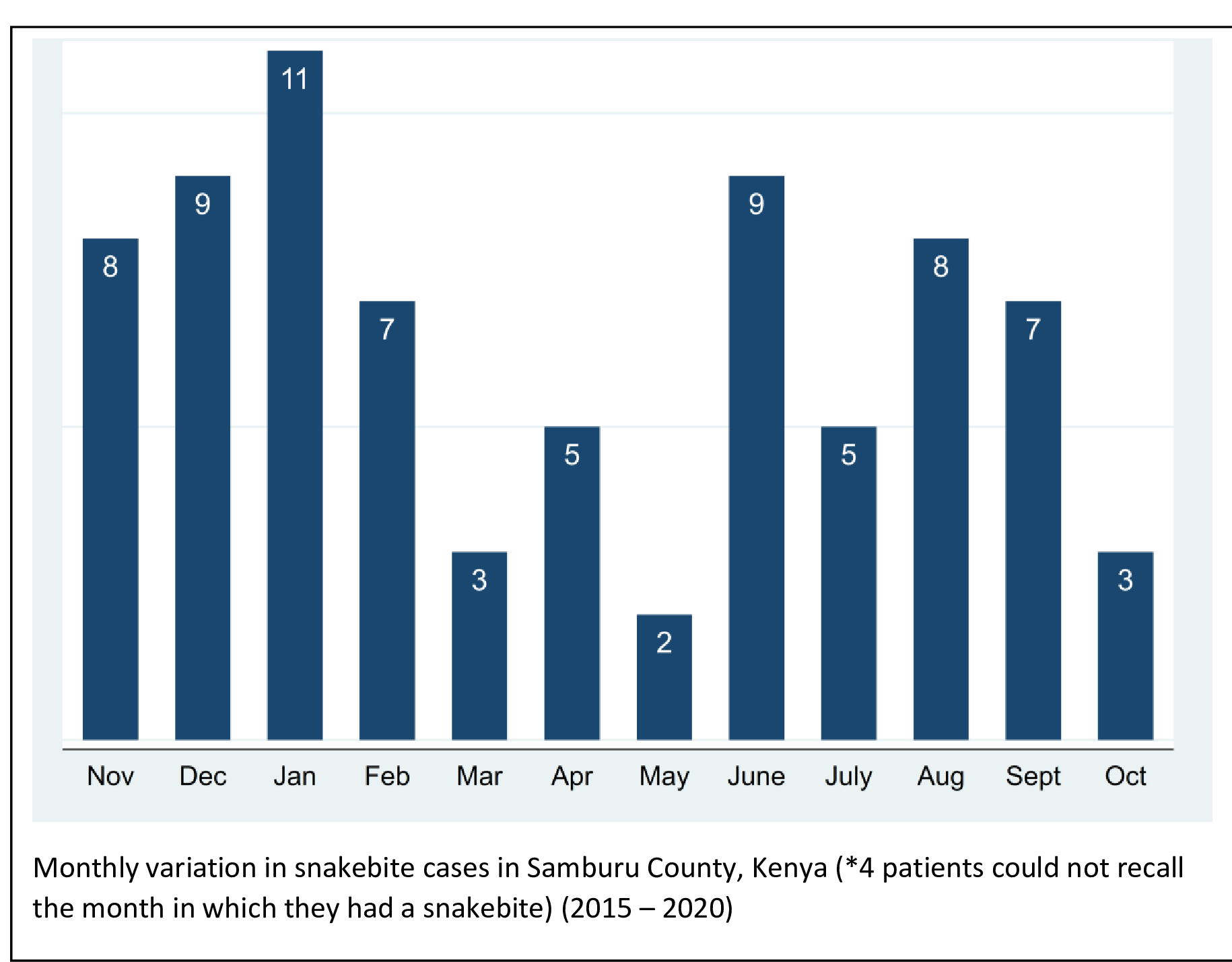

Supplement: S1 Fig — (TIF) [file pntd.0011678.s005.tif]
